# Pharmacists’ Knowledge Regarding Drug Disposal in Karbala

**DOI:** 10.3390/pharmacy7020057

**Published:** 2019-06-10

**Authors:** Khansaa A. Ibrahim Albaroodi

**Affiliations:** Pharmacy Department, Al Safwa University College, Karbala 56001, Iraq; khansaa.albaroodi@hotmail.com or khansaa.albaroodi@alsafwa.edu.iq

**Keywords:** drug disposal, pharmacist knowledge, take back program

## Abstract

**Background**: Consumers and caregivers should remove expired, or unwanted, medications to minimize the chance for misuse or accidentally using those medicines. This study investigated pharmacists’ knowledge regarding drug disposal in Karbala, Iraq. **Methods**: This study was a cross-sectional survey among pharmacists in Karbala. It was performed from December 2018 until January 2019. A standardized, 12-item, self-administered questionnaire was designed, developed and validated to assess pharmacists’ knowledge when generating pharmaceutical waste in pharmacies. **Results**: One hundred twenty-nine participants enrolled in the study. The mean age of participants was 33 ± 9.3 years—more than two-thirds (65.9%)—agreed that the return of medications to the source would be appropriate drug disposal. A good proportion of participants agreed with disposing of drugs in the trash. Further, 63.6% believe that education is the main barrier opposing the implementation of a medicine–take–back program in Iraq. **Conclusion**: Pharmacists had relatively poor knowledge regarding drug disposal methods. Health care providers (not only pharmacists) need educational courses and workshops to improve their knowledge regarding medication disposal in Iraq.

## 1. Introduction

During natural disasters, large amounts of medications are often donated. Undoubtedly lots of these pharmaceuticals save lives [1]. These pharmaceuticals may reach their expiration date and become inappropriate for use [1]. Smaller quantities of medication waste can be increased due to inadequacies in drug management and distribution, as well as lacking a routine system for drug disposal. Unsafe disposal of these unused or expired medications represent a serious problem [1]. In an increasingly urgent effort to keep these medications out of the wrong hands, pharmacies are the cornerstone for the return of unused medications. Caregivers and consumers have to reduce the possibility of accidentally or intentionally misuse these unneeded medicines in order to prevent drugs from entering our environment. The United State Food and Drug Administration has outlined some options and instructions for consideration during medication disposal—medicine–take–back (a program aimed to increase safe disposal of pharmaceuticals and to reduce pharmaceutical waste by returning unused and expired pharmaceuticals to community pharmacies or to collection depots), disposal in the trash and/or flushing in toilets [2]. If a take-back or returning-back program is not available, most unused or expired medicines can be disposed of by mixing them with an in active substance like dirt, cat litter, or used coffee grounds. Then, place it in a good sealable plastic bag to be thrown in the trash [2]. The exceptions are medicines that contain controlled substances, they should not be thrown in the trash. When there is no application for a take-back program, the Food and Drug Administration (FDA) recommended that these medications should be disposed of through flushing [2]. However, medicine and other products have been found in rivers, waterways, and groundwater—wastewater treatments are not enough to filter out these chemicals, so they are showing up in drinking water [3,4,5]. Other disposal methods include immobilization, waste encapsulation (solid medication, semi-solid medication, powder medication, liquid medication, anti-neoplastic medication, controlled substance), inertization, and landfill (old technique) [1]. Another disposal method is high-temperature incineration above (1200 °C) [1]. Incineration method had to be equipped with enough emission control. Incineration of the current stockpile of waste pharmaceuticals will be costly for the government [1]. Methods for safe disposal of pharmaceuticals pose minimal risk to public health and environment, also differ in their suitability for countries with limited resources and equipment, like Iraq. Incineration is the usual disposal method in Iraqi hospitals. However, there are other sources of drug disposal besides hospitals including drug storage facilities, private hospitals and household pharmaceutical waste. Many steps should be taken when disposing of unwanted pharmaceuticals (hospital, regional pharmacist or organizations with medical programs decide when action needs to be started). Approval of pharmaceutical disposal requires many elements like planning, funding, necessary expertise, safety and health of work teams, and categorization of the pharmaceuticals products into separate categories for that different disposal methods. Disposal options vary considerably between situations—controlled substances (e.g., narcotic medication and psychotropic medication) need tight security. 

Several studies investigated methods used for medication disposal in many different places around the world [6,7,8,9,10,11]. We performed this study to encourage safe and appropriate disposal of pharmaceuticals by communities and to bring the issue forward to the government. The important step is understanding the level of community knowledge towards this issue. This study aimed to investigate pharmacists’ knowledge regarding drug disposal in Karbala, Iraq, and to determine what barriers oppose implementation of take-back programs in Iraq from their point of view.

## 2. Materials and Methods 

This was a cross-sectional study involving pharmacists in Karbala, Iraq. It was performed from December 2018 until the end of January 2019 and included pharmacists in the government health sector (Al-Hussain General Hospital; Gyn./Obstetrics Hospital) and pharmacists working in private pharmacies at Karbala. Only 129 pharmacists responded with the questionnaire as they were given a choice to participate. Therefore, a standardized, 20-items, self-administered questionnaire designed and developed to evaluate pharmacist knowledge when generating pharmaceutical waste in pharmacies. The questionnaire was developed depending on information drawn from the literature, and a number of questions were adapted and modified from other studies regarding pharmacist knowledge on drug disposal and its effect on health and environment [1,12,13,14,15,16,17,18]. Validation was performed to determine whether the study tool measured the intended goal of this study. Appearance and content validity were assessed [19]. The questionnaire was reviewed by nine pharmacists who were faculty members with good experience in research. The suggested modifications included rephrasing questions and clarifying them. Readability testing was performed by using Microsoft Word to enhance the questionnaire’s readability [20].

The survey instrument was pretested to a sample of 20 pharmacists. At this point in the analysis, the focus was on the sufficiency of variable correlations to conduct a principal component analysis. After running a factor analysis test for the 20-item questionnaire to evaluate pharmacist knowledge on medical waste disposal and its effect on health and the environment, Kaiser-Meyer-Olkin (KMO) value was beneath the accepted limit (KMO = 0.242) [21,22]. Thus, seven variables were extracted from the analyses: participants knowledge toward proper methods of drug disposal, their knowledge regarding the existing take–back program, other barriers and participation barriers in the district applying the take–back program, participant’s knowledge of whether improper medication disposal affects the environment and whether water treatment techniques can remove most of these drugs. After reduction, the KMO became 0.619. A reliability test was performed to ensure questionnaire consistency when it was repeated under the same conditions, with internal consistency was determined by using Cronbach’s alpha [23]. Reliability analysis of the questionnaire using Cronbach’s alpha showed an internal consistency reliability of 0.660, which is satisfactory for a preliminary study, therefore reliability should be equal or above (0.60) [24]. The readability test was done by using Microsoft Word to make increment in questionnaire validity [20]. The questionnaire was readable by respondents and was pretested on a convenient sample of 129 participants. Participants were able to answer the questions within 5 min. The questionnaire was valid and reliable for the evaluation of pharmacists’ knowledge regarding medication disposal and barriers opposing the application of take–back programs in Iraq. The questionnaire contains 12 questions, which included the following information:

1. Section 1: Participants demographic data, which included age, gender, official title, and experience.
2. Section 2: Participants knowledge toward proper methods of drug disposal and barriers opposing the implementation of take–back programs in Iraq. Moreover, it assessed their agreement that scientific and educational courses, workshops and symposia can increase awareness and experience of those working in the health field.

### Statistical Analyses

The collected data had been analyzed using SPSS (version 18.0) software package (SPSS Inc., Chicago, IL, USA). Almost all questions had been calculated as proportions and percentages as they are categorical variables. Respondents who participated in the pilot study were excluded from the final study analyses.

## 3. Results

Approximately 150 of the surveys were distributed among pharmacists in Karbala and only 129 of them were filled out. One hundred and twenty-nine participants enrolled in the study they were included in the analysis. The mean age for the participants was 33 ± 9.3 years, and the majority of the participants 128 (99.2%) were working in the Ministry of Health with experience 8.7 ± 8.9 years. Table 1 shows the participants’ knowledge related to drug disposal. A good proportion agreed with drug disposal via the trash. However, around two-thirds disagreed with drug disposal through the sink and/or incineration. On the other hand, 65.9% of them agreed to return medication to the source (drug store or company). 

Two-thirds of the participants agreed that liquid drugs can be disposed of via the sink. However, an almost equal proportion of participants agreed that the trash is a suitable disposal method for fentanyl patches (33.3%), while another proportion (28.7%) agreed to return them to the source. About one-third of the participants agreed that trash disposal would be appropriate for inhaler products. The trash disposal method was thought to be appropriate to dispose semi-solid pharmaceutical products in 41.9% of participants, Table 2.

About two-thirds of the participants acknowledged the take-back program. A large proportion of the participants (77.5%) did not agree that time is a barrier restricting the application of a take-back program. However, two-thirds though that education on the program was the main barrier, Table 3. 

## 4. Discussion

A pharmacist’s role has moved from compounding and dispensing medications towards providing patients with care. This has created an urgent need to ameliorate pharmacist knowledge regarding medication use and disposal. The FDA and WHO outlined their guidelines for proper drug disposal and this research evaluated pharmacists’ knowledge regarding these guidelines [1,2]. Our results show that two-thirds of participants agreed that liquid drugs can be disposed of via a sink. However, disposal of liquid medications via the sink would not always be proper for all liquid medications [1]. About one-third of participants agreed that the trash would be a suitable disposal method for fentanyl patches (33.3%). However, the WHO mentioned that these controlled medicines should be disposed of by encapsulation or inertization [1]. About one-third of the study participants agreed that the trash disposal method would be appropriate for inhaler products. Although guidelines stated that they could be dangerous if punctured or thrown into a fire or incinerator [2], so they should be dispersed among municipal solid wastes or disposed of in a landfill [1]. Our results show that the trash disposal method was thought to be appropriate for the disposal of semi-solid pharmaceutical products by 41.9% of the participants, despite the WHO recommendation of encapsulation for semi-solid waste [1]. More than two-thirds of the study participants agreed that returning medications to the source would be the best way of disposal. However, a study in Kabul aimed to determine disposal practices of medications among the general public, they found that 77.7% of study participants discarded medications in the trash [10]. 

The majority of study respondents consider the government responsible for creating awareness for proper disposal, and felt that improper medication disposal can affect health and environment [10]. Another study by Braund and colleagues aimed to determine the proportion of unused medications in New Zealand that had not returned to the pharmacy for disposal [9]. The study was completed by 452 respondents. More than half of them had unwanted medications and 13–24% of their unused medications returned to the pharmacist [9]. A cross-sectional study investigated people’s behavior with respect to the pharmaceutical products disposal. Tt aimed to identify the best way of education regarding safe disposal of medication [11]. They found that among 50 participants, about 79% of participants disposed of unused medication by household waste, while a small proportion (1.70%) of them returned unused medication to a pharmacy and 78.6% of them expressed an interest in receiving information concerning the correct disposal method of medication [11]. However, the majority of our study participants (85.3%) agreed that educational courses on a take–back program can improve knowledge regarding medication disposal. Another study in Kuwait measured attitude and practice of patients regarding safe disposal of medication [6]. The most common disposal procedures were to throw medications in the trash (76.5%) or flush them down (11.2%). However, about half of them (54.0%) thought that taking medicines to pharmacies for ensuring safe disposal would be favorable [6]. Moreover, a study in Madigan evaluated patient’s practices and beliefs concerning medications disposal [7]. They found that more than half of the patients stored expired or unused medications in their houses, and more than half of them flushed them down a drain. Only 22.9% of study participants reported that they had returned drugs to the pharmacy for more suitable disposal. Less than 20% of them had ever been given advice regarding medication disposal by a healthcare provider [7], suggesting that there is an important role for patient education on the proper medication disposal [7]. In Nigeria, basic education on the appropriate disposal of medicines needed, as unused medications are not returned to the pharmacies for appropriate disposal as in the developed countries [8].

More than two-thirds of these study participants believe that education is the main barrier opposing the implementation of take-back programs in Iraq. Medication disposal is an important issue because it has an impact on our lives and ecosystem. However, this issue has never been discussed in such a way that all healthcare providers should have a role in the process and be educated on how to minimize improper disposal. Usually, the government healthcare sector assumes this role. In Iraq, which is a developing country with a lack of knowledge and awareness regarding the appropriate methods for medication disposal, an important step is improving knowledge regarding the disposal of unused pharmaceuticals. This study was performed with the hope that it could become a cornerstone in this area. However, this study is limited by the small number of participants.

## 5. Conclusions

In conclusion, pharmacists had relatively poor knowledge regarding drug disposal methods. Iraqi healthcare providers (not only pharmacists) need educational courses and workshops to improve their knowledge regarding medication disposal. Further, the Iraqi government needs to take many steps to ensure that medication disposal and treatment create safe waste that does not harm the population or environment.

## Figures and Tables

**Table 1:** Pharmacist’s knowledge regrading proper methods of drug disposal.

Item	Frequency (%)
**Gender**	
Male	70 (54.3)
Female	59 (45.7)
Total	129 (100)
**Drug Disposal via Trash**	
Yes	60 (46.5)
No	69 (53.5)
Total	129 (100)
**Drug Disposal via Sink**	
Yes	45 (34.9)
No	84 (65.1)
Total	129 (100)
**Drug Returned to Source (Drug Store or Company)**	
Yes	85 (65.9)
No	44 (34.1)
Total	129 (100)
**Drug Disposal via Incineration**	
Yes	47 (36.4)
No	82 (63.6)
Total	129 (100)

**Table 2:** Appropriateness of common methods used for drug disposal for a special drug.

Item	Frequency (%)
**Liquid Drug Disposal**	
Trash	21 (16.3)
Sink	78 (60.5)
Return to source	25 (19.4)
Incineration	3 (2.3)
Others	1 (0.8)
Missing data	1 (0.8)
Total	129 (100)
**Fentanyl Patches Disposal**	
Trash	43 (33.3)
Sink	3 (2.3)
return to source	37 (28.7)
Incineration	40 (31)
Others	2 (1.6)
Missing data	4 (3.1)
Total	129 (100)
**Inhaler Products Disposal**	
Trash	38 (29.5)
Sink	2 (1.6)
return to source	55 (42.6)
Incineration	30 (23.3)
Others	2 (1.6)
Missing data	2 (1.6)
Total	129 (100)
**Semi Solid Disposal**	
Trash	54 (41.9)
Sink	3 (2.3)
return to source	43 (33.3)
Incineration	28 (21.7)
Missing data	1 (0.8)
Total	129 (100)

**Table 3:** Applying a take-back program: Its barriers and benefits from educational courses regarding a take-back program.

Item	Frequency (%)
**Knowledge Regarding Take Back Program**	
Yes	76 (58.9)
No	52 (40.3)
Total	128 (99.2)
Missing data	1 (0.8)
Total	129 (100)
**Take Back Program Education Barrier**	
Yes	82 (63.6)
No	47 (36.4)
Total	129 (100)
**Take Back Program Cost Barrier**	
Yes	57 (44.2)
No	72 (55.8)
Total	129 (100)
**Take Back Program Time Barrier**	
Yes	29 (22.5)
No	100 (77.5)
Total	129 (100)
**Educational Courses on Take Back Program**	
Agree	110 (85.3)
no idea	10 (7.8)
Disagree	9 (7)
Total	129 (100)
